# Protective Effect of Salidroside from *Rhodiolae Radix* on Diabetes-Induced Oxidative Stress in Mice

**DOI:** 10.3390/molecules16129912

**Published:** 2011-12-01

**Authors:** Fenglin Li, Hong Tang, Furen Xiao, Jingli Gong, Yong Peng, Xiangle Meng

**Affiliations:** 1 Key Laboratory of Metastable Materials Science and Technology, College of Materials Science and Engineering, Yanshan University, Qinghuangdao, 066004, China; Email: swgclfl@163.com; 2 Department of Bioengineering, Jilin Agricultural Science and Technology College, Jilin, 132101, China; Email: 591142473@qq.com; 3 Department of Endocrinology, Longhua Hospital, Shanghai University of Traditional Chinese Medicine, Shanghai, 200032, China; Email: lhhong_tang@qq.com; 4 College of Environment and Chemical Engineering, Yanshan University, Qinghuangdao, 066004, China; Email: py81ysu@126.com; 5 Department of Pharmacy, The First Affiliated Hospital of Henan College of Traditional Chinese Medicine, Zhengzhou, 450000, China; Email: mxiangle@yahoo.cn

**Keywords:** oxidative stress, diabetes mellitus, salidroside, hypoglycemic, mice

## Abstract

It has been confirmed that diabetes mellitus (DM) carries increased oxidative stress. This study evaluated the effects of salidroside from *Rhodiolae Radix *on diabetes-induced oxidative stress in mice. After induction of diabetes, diabetic mice were administered daily doses of 50, 100 and 200 mg/kg salidroside for 28 days. Body weights, fasting blood glucose (FBG), serum insulin, TC (total cholesterol), TG (triglyceride), malondialdehyde (MDA), superoxide dismutase (SOD), glutathione peroxidase (GPx) and catalase (CAT) were measured. Results showed that salidroside possessed hypoglycemic activity and protective effects against diabetes-induced oxidative stress, which could significantly reduce FBG, TC, TG and MDA levels, and at same time increase serum insulin levels, SOD, GPx and CAT activities*.* Therefore, salidroside should be considered as a candidate for future studies on diabetes.

## 1. Introduction

Diabetes mellitus (DM) is a chronic disease caused by inherited and/or acquired deficiency in production of insulin by the pancreas, or by the ineffectiveness of the insulin produced. Such a deficiency results in increased concentrations of glucose in the blood, which in turn damage many of the body’s systems, in particular the blood vessels and nerves [1]. Chronic hyperglycemia during diabetes causes glycation of body proteins that in turn leads to secondary complications affecting eyes, kidneys, nerves and arteries [2]. There are two types of diabetes, namely type 1 and type 2. Type 1, insulin-dependent diabetes mellitus (IDDM), in which the body does not produce any insulin, most often occurs in children and young adults. Type 1 diabetes accounts for 5–10% of diabetes. Type 2, noninsulin-dependent diabetes mellitus (NIDDM), in which the body does not produce enough, or properly use, insulin, is the most common form of the disease, accounting for 90–95% of diabetes [3]. The world prevalence of diabetes among adults (aged 20–79 years) in 2010 will be 6.4%, affecting 285 million adults, and will increase to 7.7%, and 439 million adults by 2030. Between 2010 and 2030, there will be a 69% increase in numbers of adults with diabetes in developing countries and a 20% increase in developed countries [4,5]. In recent years, many studies have confirmed that DM carries increased oxidative stress [6]. Hyperglycemia can increase oxidative stress due to increased mitochondrial production of the superoxide anion nonenzymatic glycation of proteins and glucose autoxidation [7,8,9]. Free fatty acid (FFA) levels, which are elevated in diabetes and insulin resistance, may also contribute to the increased production of reactive oxygen species (ROS) due to increased mitochondrial uncoupling and ß-oxidation [10,11] Several studies have shown that antioxidants could be useful in preventing or attenuating the adverse effects of chronic hyperglycemia [12,13,14].

**Figure 1:**
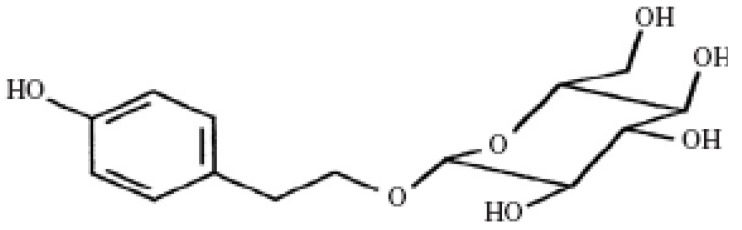
Molecular structure of salidroside.

The modern drugs, including insulin and other oral hypoglycemic agents such as biguanides, sulphonylureas, α-glycosidase inhibitors, control the blood glucose level as long as they are regularly administered but they may also produce a number of undesirable effect [15,16,17,18]. Hence there is a need to search for newer anti-diabetic agents that have high therapeutic efficacy with minimum side effects. Over the past few decades, Traditional Chinese Medicine has been confirmed to be effective and promising in the therapy of diabetes and its complications with few undesirable effects compared with the modern drugs [19,20,21,22]. Based on a large number of chemical and pharmacological research works, numerous bioactive compounds for diabetes have been found in Chinese medicinal plants [3]. The roots of *Rhodiola sachalinensis* A. BOR. (Gao-shan-hong-jing-tian in Chinese), *Rhodiolae Radix*, have been widely used as a hemostatic, antitussive, tonic, and endermic liniment for burns and contusions in Traditional Chinese Medicine [23,24,25]. Moreover, in animal models, *Rhodiolae Radix* extract has been reported to have a very good effectiveness in the control of blood glucose [26,27]. Salidroside, 2-(4-hydroxyphenyl)ethyl-β-D-glucopyranoside, a compound with a phenol glycoside chemical structure (Figure 1), is isolated from the *Rhodiolae Radix* as one of its main active ingredients and is thought responsible for all the documented pharmacological effects of the medicinal plant. Previous studies demonstrated that salidroside has important bioactivities including anti-fatigue, anti-inflammatory, anticancer, antioxidant, anti-aging, hepatoprotective effects, *etc.* [28,29,30,31,32]. Besides, Tong *et al.* indicated that salidroside has a significant effect on glucose, fat and amino acid metabolism and can improve the acrobic capacity. Another study also proves that salidroside could stimulate glucose uptake in differentiated L6 rat myoblast cells [33]. However, few studies have examined the hypoglycemic activity of salidroside. The present study was undertaken to evaluate the effects of salidroside on diabetes-induced oxidative stress in mice.

## 2. Results and Discussion

### 2.1. Effect of Salidroside Administration on Body Weight in Mice

Table 1 shows the effect of salidroside administration on body weight in mice. Before the experiment, body weights between groups were not significantly different, but after 7 days, body weighs of normal controls (NC) were significantly increased when compared with the diabetic groups (*P < 0.05*). However, body weight changes were not significant between the diabetic control (DC), low dose salidroside (50 mg/kg) treatment (DLT), middle dose salidroside (100 mg/kg) treatment (DMT) and high dose salidroside (200 mg/kg) treatment (DHT) groups. After 28 days, NC group had an average increased body weight of 27.7%. When compared with DC group, body weights were significantly increased (*P < 0.05*) in DLT, DMT and DHT groups.

**Table 1:** Effect of salidroside administration on body weight in mice.

Groups	0 days	7 days	14 days	21 days	28 days
NC	24.41 ± 2.16	27.84 ± 2.47 ^b^	28. 97 ± 2.68 ^b^	30.64 ± 1.97 ^b^	31.16 ± 2.53 ^b^
DC	24.17 ± 1.48	22.39 ± 3.24 ^a^	22.81 ± 3.65 ^a^	23.76 ± 2.43 ^a^	23.31 ± 3.15 ^a^
DLT,	23.86 ± 2.34	23.25 ± 2.17 ^a^	24.29 ± 2.87 ^a^	28.57 ± 1.94 ^b^	29.14 ± 2.47 ^b^
DMT	23.79 ± 1.67	23.16 ± 1.49 ^a^	25.25 ± 2.12 ^a^ ^b^	28.41 ± 2.45 ^b^	29.39 ± 2.13 ^b^
DHT	24.33 ± 1.95	23.48 ± 1.83 ^a^	26.26 ± 2.08 ^b^	29.33 ± 1.87 ^b^	30.51 ± 2.52 ^b^

Data were presented as means ± SD. NC: normal control, DC: diabetic control; DLT: low dose salidroside (50 mg/kg) treatment, DMT: middle dose salidroside (100 mg/kg) treatment, DHT: high dose salidroside (200 mg/kg) treatment. ^a^ *P < 0.05* as compared with the NC group; ^b^ *P < 0.05* as compared with the DC group.

The pancreas is the primary organ involved in sensing the organism’s dietary and energetic states via glucose concentration in the blood and in response to elevated blood glucose, insulin is secreted [34]. Alloxan, induces “chemical diabetes” in a wide variety of animal species by damaging the insulin secreting cells of the pancreas. This damages a large number of β-cells, resulting in a decrease in endogenous insulin release, which paves the ways for the decreased use of glucose by the tissues [35]. Alloxan-induced diabetes is characterized by severe loss in body weights and similar results also were observed in the present study. This loss of body weights could be due to, dehydration and catabolism of fats and protein [36]. Previous reports show that protein synthesis is decreased in all tissues due to decreased production of ATP and absolute or relative deficiency of insulin [37]. In this study, the diabetic control group showed significant loss of body weight and the salidroside treatment groups showed significant prevention of this loss in body weights, which might be as a result of its ability to reduce hyperglycemia. It is very meaningful that the prevention effects could be applied to clinical diabetics who lose weight.

### 2.2. Effect of Salidroside Administration on Fasting Blood Glucose Levels in Mice

**Figure 2:**
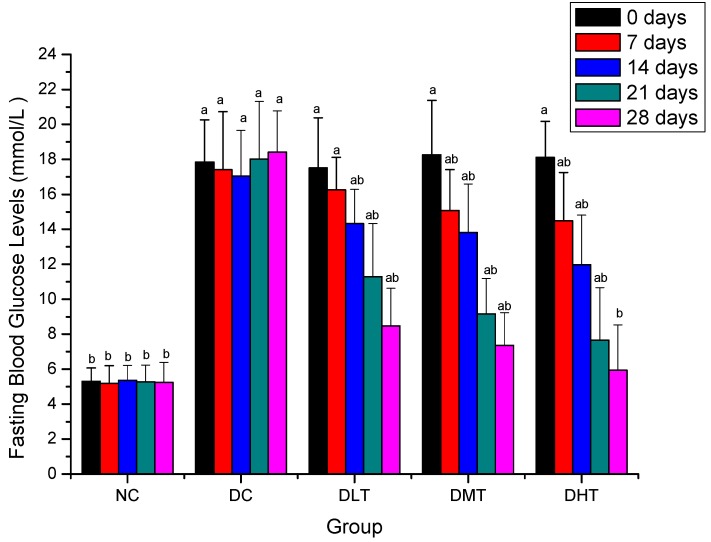
Effect of salidroside administration on fasting blood glucose levels in mice. Data were presented as means ± SD. NC: normal control, DC: diabetic control; DLT: low dose salidroside (50 mg/kg) treatment, DMT: middle dose salidroside(100 mg/kg) treatment, DHT: high dose salidroside(200 mg/kg) treatment. ^a^ *P < 0.05* as compared with the NC group. ^b^ *P < 0.05* as compared with the DC group.

Figure 2 shows the effect of salidroside administration on fasting blood glucose levels in mice. FBG levels of NC group maintained constant during the experimental period and were significantly decreased when compared with the diabetic groups before experiment (*P < 0.05*). After 14 days, FBG levels of DLT, DMT and DHT groups were significantly decreased when compared with DC group (*P < 0.05*) in a dose-dependent manner and the strongest effect was seen with 200 mg/kg dose. After 28 days, FBG levels changes were not significant between the NC and DHT groups.

It is known that effective control of the blood glucose level is a key step in preventing or reversing diabetic complications and improving the quality of life in diabetic patients [38,39,40]. The hypoglycemic activity of salidroside in alloxan-induced diabetic mice has been indicated here by estimation of fasting blood glucose levels, as the important basal parameter for monitoring of diabetes. The possible mechanism of this hypoglycemic activity may be by potentiation of pancreatic secretion of insulin from β-cell of islets or due to enhanced transport of blood glucose to peripheral tissue [41]. Therefore, the effect of salidroside on serum insulin levels in alloxan-induced diabetic mice was investigated.

### 2.3. Effect of Salidroside Administration on Serum Insulin Levels in Mice

**Figure 3:**
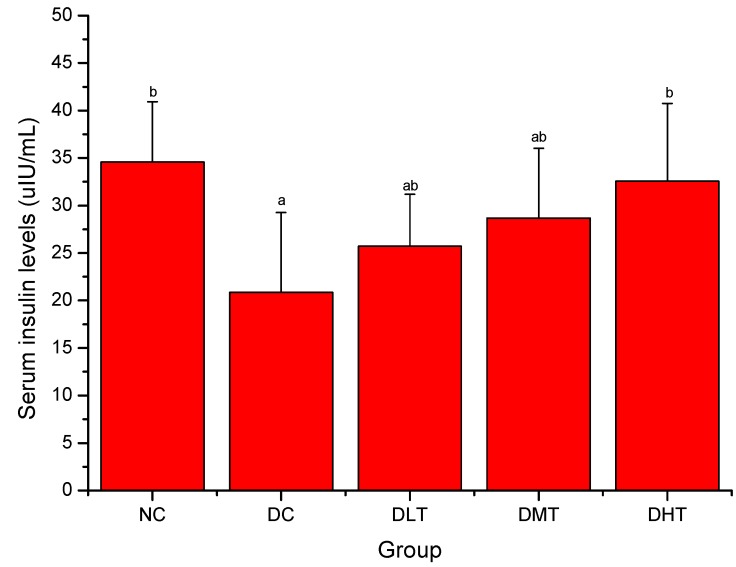
Effect of salidroside administration on serum insulin levels in mice. Data were presented as means ± SD. NC: normal control, DC: diabetic control; DLT: low dose salidroside (50 mg/kg) treatment, DMT: middle dose salidroside (100 mg/kg) treatment, DHT: high dose salidroside (200 mg/kg) treatment. ^a^ *P < 0.05* as compared with the NC group. ^b^ *P < 0.05* as compared with the DC group.

Figure 3 shows the effect of salidroside administration on serum insulin levels in mice. Serum insulin levels of DC group were significantly decreased when compared with the NC groups (*P < 0.05*). After 28 days, DLT, DMT and DHT groups showed a dose-dependent increase in serum insulin levels compared with the DC group (*P < 0.05*) and the strongest effect was seen with 200 mg/kg dose.

Results of the current study showed that serum insulin levels have been increased significantly in diabetic mice after the salidroside supplementation. The increment of serum insulin levels might be due to the renewal of beta cells in the pancreas or the recovery of partially destroyed β-cells [42,43].

### 2.4. Effect of Salidroside Administration on Blood Lipid Levels in Mice

**Figure 4:**
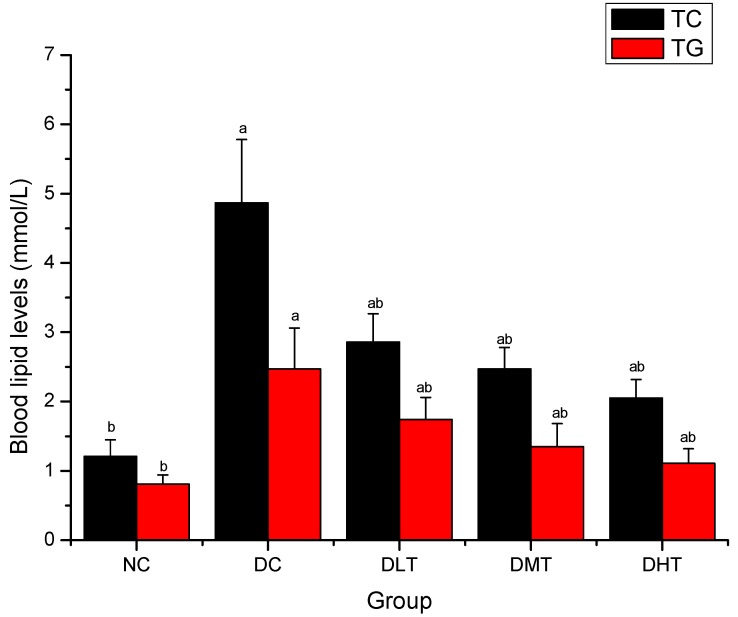
Effect of salidroside administration on blood lipid levels in mice. Data were presented as means ± SD. NC: normal control, DC: diabetic control; DLT: low dose salidroside (50 mg/kg) treatment, DMT: middle dose salidroside (100 mg/kg) treatment, DHT: high dose salidroside (200 mg/kg) treatment. ^a^ *P < 0.05* as compared with the NC group. ^b^ *P < 0.05* as compared with the DC group.

Figure 4 shows the effect of salidroside administration on blood lipid levels in mice. TC and TG levels of DC group were significantly increased when compared with the NC groups (*P < 0.05*). After 28 days, TC and TG levels of DLT, DMT and DHT groups were significantly decreased when compared with the DC groups (*P < 0.05*).

Diabetes is frequently accompanied by multiple metabolic abnormalities, including dyslipidemia, which increases the risk for cardiovascular disease. Reduction in TG and TC through dietary or drug therapy has been found beneficial in preventing diabetic complications as well as improving lipid metabolism in diabetic patients [44,45,46]. In this study, TG and TC levels have been decreased significantly in diabetic mice after the salidroside supplementation. These effects might be due to low activity of cholesterol biosynthesis enzymes and/or low level of lipolysis, which are under the control of insulin [47].

### 2.5. Effect of Salidroside Administration on Antioxidant Enzymes Activities and MDA Levels in Kidney and Liver in Mice

Table 2 and Table 3 show the effect of salidroside administration on antioxidant activities enzymes and MDA levels in kidney and liver in mice. There appears to be a significant increase (*P < 0.05*) in the MDA levels associated with the diminution of the SOD, GPx and CAT activities of DC group in comparison with NC group. After 28 days, SOD, GPx and CAT activities of DLT, DMT and DHT groups were higher and MDA levels were lower than that of DC group (*P < 0.05*).

It has been confirmed that diabetes mellitus carries increased oxidative stress. Diabetics and experimental animal models exhibit high oxidative stress due to persistent and chronic hyperglycemia, which depletes the activity of antioxidative defense system resulting in elevated levels of oxygen free radicals [48,49,50].

**Table 2:** Effect of salidroside administration on antioxidant enzymes activities and MDA levels in kidney in mice.

Groups	Kidney			
	SOD (U/mg protein)	GPx (U/mg protein)	CAT (U/mg protein)	MDA (nmol/mg protein)
NC	10.87 ± 1.08 ^b^	7.43 ± 0.98 ^b^	14.29 ± 2.07 ^b^	1.57 ± 0.16 ^b^
DC	4.64 ± 0.95 ^a^	4.02 ± 0.67 ^a^	8.23 ± 0.96 ^a^	4.11 ± 0.12 ^a^
DLT	6.99 ± 1.21 ^a^ ^b^	7.11 ± 1.24 ^b^	13.94 ± 1.98 ^b^	2.84 ± 0.27 ^a^ ^b^
DMT	8.03 ± 0.84 ^a^ ^b^	7.93 ± 0.85 ^b^	15.31 ± 1.87 ^b^	2.36 ± 0.31 ^a^ ^b^
DHT	8.16 ± 1.35 ^a^ ^b^	8.26 ± 1.17 ^b^	18.49 ± 2.23 ^a^ ^b^	1.79 ± 0.25 ^b^

Data were presented as means ± SD. NC: normal control, DC: diabetic control; DLT: low dose salidroside (50 mg/kg) treatment, DMT: middle dose salidroside (100 mg/kg) treatment, DHT: high dose salidroside (200 mg/kg) treatment. ^a^ *P < 0.05* as compared with the NC group; ^b ^*P < 0.05* as compared with the DC group.

**Table 3:** Effect of salidroside administration on antioxidant enzymes activities and MDA levels in liver in mice.

Groups	Liver			
	SOD (U/mg protein)	GPx (U/mg protein)	CAT (U/mg protein)	MDA (nmol/mg protein)
NC	12.08 ± 2.19 ^b^	4.14 ± 0.54 ^b^	19.16 ± 2.81 ^b^	6.61 ± 0.47 ^b^
DC	6.16 ± 0.98 ^a^	1.12 ± 0.26 ^a^	11.43 ± 2.35 a	9.27 ± 0.84 ^a^
DLT,	8.95 ± 1.17 ^a^ ^b^	3.47 ± 0.31 ^b^	15.60 ± 1.91 ^a^ ^b^	8.89 ± 0.58 ^a^
DMT	10.99 ± 1.64 ^b^	4.09 ± 0.48 ^b^	18.47 ± 2.13 ^b^	7.26 ± 0.67 ^b^
DHT	11.32 ± 2.25 ^b^	4.63 ± 0.41 ^b^	20.24 ± 1.68 ^b^	7.01 ± 0.49 ^b^

Data were presented as means ± SD. NC: normal control, DC: diabetic control; DLT: low dose salidroside (50 mg/kg) treatment, DMT: middle dose salidroside (100 mg/kg) treatment, DHT: high dose salidroside (200 mg/kg) treatment. ^a^ *P < 0.05* as compared with the NC group; ^b^ *P < 0.05* as compared with the DC group.

Attack by high levels of free radicals and simultaneous decrease of the expression of antioxidant enzymes, may enhance membranes’ susceptibility to lipid peroxidation and lead to pancreatic β-cell dysfunction, as well as other cellular organelle damage [51,52]. The increase in lipid peroxide levels is one of the most important contributing factors in the development of diabetes-related complications [35]. Thus, the ideal antidiabetic drug should have hypoglycaemic effects. Besides, it may be better to have some antioxidative properties too.

Earlier studies had reported a decrease in the activities of antioxidant enzymes (SOD, CAT and GPx) in the kidney and liver in diabetic animal [53,54]. Similar results also were observed in the present study and the administration of salidroside significant increased SOD, GPx and CAT activities in the kidney and liver of diabetic mice. Lipid peroxidation products such as MDA are generated under high levels of un-scavenged free radicals and may bring about protein damage and inactivation membrane bound enzymes, so they play an important role in pancreatic damage associated with diabetes [52]. In this study, MDA levels have been decreased significantly in the kidney and liver in diabetic mice after the salidroside supplementation. This means that the salidroside may reduce reactive oxygen free radicals or improve the activities of antioxidant enzymes, prevent (or reduce) lipid peroxidation. Therefore, salidroside showed in our study it had a direct and indirect preventive and protective effect in diabetes by decreasing oxidative stress.

## 3. Experimental

### 3.1. Chemicals and Reagents

Salidroside (99% purity) was purchased from Chengdu Sikehua Biotechnology Co., Ltd. (Chengdu, China). A glucose analyzer (GT-1640) and glucose check strips were purchased from Arkray Inc. (Japan). Alloxan was purchased from Sigma Chemical Co. (USA). Reagents for TC (total cholesterol) and TG (triglyceride) were purchased from Beijing Chengxinde Biochemistry Reagent Co. (Beijing, China). Reagents for superoxide dismutase (SOD), glutathione peroxidase (GPx), catalase (CAT) and malondialdehyde (MDA) were purchased from Nanjing Jiancheng Biochemistry Reagent Co. (Nanjing, China). Reagents for serum insulin were purchased from Beijing Beifang Pharmaceutical Co. (Beijing, China). All of the other chemicals and reagents were standard commercially available biochemical quality. Water was purified with a Milli-Q purification system and was used to prepare all solutions.

### 3.2. Animals

Male mice of original Kun-ming strain, weighing 18–22 g, were used for the study. All experiments were performed in accordance with the Guide for the Care and Use of Laboratory Animals of the Chinese National Institutes of Health. The animals were obtained from the Animal Department of the Beijing Institute of Traditional Medical and Pharmaceutical Sciences (Beijing, China) and were housed in a room maintained at 23 ± 2 °C with relative air humidity of 45% to 55% on a 13-hour light/11-hour dark cycle. Mice were provided a standard laboratory chow and water *ad libitum*. The approval of this experiment was obtained from the Institutional Animal Ethics Committee of Yanshan University (Qinghuangdao, China).

### 3.3. Preparation of Diabetic Mice

The mice were adapted to diet and environment for one week before the experiment began. After a 24-hour fasting, the mice were induced with a single injection of 4% alloxan prepared freshly at a dose of 200 mg/kg bw. Diabetes was confirmed by the determination of tail vein blood glucose levels on the third day after administration of alloxan. Mice having blood glucose levels greater than 11.1 mmol/L were considered diabetic and were used for the study [55].

### 3.4. Experimental Design

After confirmation of the diabetic state, normal and alloxan-induced diabetic mice were randomly divided into five groups of ten per group.

Group I: normal control (NC), normal mice were allowed to free access to a normal diet and treated daily with distilled water (5 mL/kg) by gavage using a 20 gauge feeding needle for 28 days.

Group II: diabetic control (DC), diabetic mice were allowed to free access to a normal diet and treated daily with distilled water (5 mL/kg) by gavage using a 20 gauge feeding needle for 28 days.

Group III: low dose salidroside (50 mg/kg) treatment (DLT), diabetic mice were put on a normal diet and treated daily with 50 mg/kg of salidroside by gavage using a 20 gauge feeding needle for 28 days.

Group IV: middle dose salidroside (100 mg/kg) treatment (DMT), diabetic mice were put on a normal diet and treated daily with 100 mg/kg of salidroside by gavage using a 20 gauge feeding needle for 28 days.

Group V: high dose salidroside (200 mg/kg) treatment (DHT), diabetic mice were put on a normal diet and treated daily with 200 mg/kg of salidroside by gavage using a 20 gauge feeding needle for 28 days.

This selection of doses was based on preliminary experiments, wherein, doses of 50–200 mg/kg was tried and confirmed to be suitable and effective in test mice. Fasting blood glucose (FBG) levels were measured for once every week. Blood was collected from tip of the tail vein (starting from 9:00 a.m.) after a 12- to 14-hour overnight fast. At the same time, body weights of mice were measured using a balance. On the last day of experiment, the mice were deprived of food overnight and sacrificed by cervical dislocation. Blood was collected in polystyrene tubes without the anticoagulant. Serum was immediately separated by centrifugation at 3,000 rpm at room temperature for 10 min. Samples were stored at −20 °C for the assay of TC, TG and serum insulin. 

The kidney and liver were carefully removed and homogenized in ice-cold 0.15 M Tris-KCl buffer (pH 7.4) to yield a 10% (w/v) homogenate. The latter was next subjected to high-speed centrifugation for 30 min at 4 °C. The resulting supernatant was used as such for assaying GPx, SOD, CAT activities and MDA levels.

### 3.5. Statistical Analysis

All results are expressed as mean ± S.D. for ten mice in each group. To determine the effect of treatment, data were analyzed using one-way ANOVA repeated measures. *p*-values of less than 0.05 were regarded as significant. Significant values were assessed with Duncan’s multiple range test. Data were analyzed using the statistical package “SPSS 12.0 for Windows”.

## 4. Conclusions

In conclusion, the present investigation showed that salidroside possess hypoglycemic activity and exert protective effects in experimental diabetes, possibly by reducing diabetes-induced oxidative stress and hence protects organism from oxidative damage and dyslipidemia. This may be attributed to its antioxidative potential. Therefore, salidroside should be considered as a candidate for future studies on diabetes.
